# The effects of isolated game elements on adherence rates in food response inhibition training

**DOI:** 10.1098/rsos.241657

**Published:** 2024-12-11

**Authors:** Alexander MacLellan, Charlotte R. Pennington, Natalia Lawrence, Samuel J. Westwood, Andrew Jones, Anna Slegrova, Beatrice Sung, Louise Parker, Luke Relph, Jessica O. Miranda, Maryam Shakeel, Elizavet Mouka, Charlotte Lovejoy, Chaebin Chung, Sabela Lash, Yusra Suhail, Mehr Nag, Katherine S. Button

**Affiliations:** ^1^ Department of Psychology, University of Bath, Bath BA2 7AY, UK; ^2^ School of Psychology, Aston University, Birmingham B4 7ET, UK; ^3^ Department of Psychology, University of Exeter, Exeter EX4 4QG, UK; ^4^ Department of Psychology, Kings College London, London WC2R 2LS, UK; ^5^ School of Psychology, Liverpool John Moores University, Liverpool L2 2QP, UK

**Keywords:** cognitive training, eating behaviour, mHealth, behaviour change, obesity

## Abstract

Food response inhibition training (food-RIT) is found to aid weight loss and reduce snacking of foods high in sugar, salt and fat. However, these interventions suffer from a lack of adherence, with gamification proposed as a solution to increase engagement. The effect of gamification is unclear, however, with a lack of research investigating the effects of single game elements in improving adherence to interventions. This study investigates whether isolated game elements (social or feedback) improve adherence, engagement and effectiveness of food-RIT compared to a standard non-gamified intervention. Two hundred and fifty-two participants (169 female) were randomly assigned to either non-gamified F-RIT, a training gamified with feedback elements or a training gamified with social elements. Participants completed measures of snacking frequency and food evaluation before and after a 14-day training period, with adherence and motivation recorded during this time. There were no significant effects of adding either feedback or social gamification elements on training adherence, motivation or effectiveness. There was no meaningful support for adding isolated game elements to food-RIT to improve intervention adherence, raising questions about the magnitude of simple gamification effects. Future research may benefit from systematically assessing the combined effects of multiple gamification elements.

## 1. Introduction

Poor diet is recognized as one of the leading causes of premature mortality [1], and though attempting to reduce calorific intake by dieting is common, it is often unsuccessful [2]. This may be due to prolonged calorie deficit increasing the reward value of high-calorie food [3]. Foods high in fat, sugar and salt (HFSS) generate strong reward responses that people susceptible to overeating can struggle to inhibit [4]. Food-specific response inhibition training (F-RIT) targets these automatic responses by training participants to inhibit motor responses to HFSS foods in computerized tasks (e.g. go/no-go tasks). Such training has been shown to reduce participants’ consumption of the targeted unhealthy food in lab-based studies [5], facilitate short-term weight loss in field studies [6,7] and reduce the palatability of HFSS food items [8,9].

F-RIT involves training participants to inhibit motor responses to unhealthy foods, pairing cues to withhold a motor response with HFSS stimuli, often in comparison with healthy food stimuli and non-food related controls [5]. The mechanisms by which these interventions operate are still uncertain, though recent reviews propose that the learned motor response inhibition in response to HFSS foods conflicts with the reward responses elicited by the appetitive unhealthy food, which is resolved by a devaluation of the food stimulus [4,10,11]. In recent reviews, the effect of F-RIT on food devaluation has been demonstrated [12] and is supported by neuroimaging studies that find reductions in activity in reward- and attention-related regions of the brain [13,10]. Though there is promising evidence of the potential benefits of F-RIT, there are questions about adherence rates to computerized cognitive training delivered outside of the lab [14,15], as inconsistent usage of this intervention or stopping altogether can reduce the efficacy of training [16]. Supporting this, Chen *et al*. [17] found the effect of F-RIT on food choices to be reduced within weeks, suggesting the need for regular training.

RIT relies on the repetition of motor responses to similar stimuli hundreds of times over the course of an intervention, and engagement levels can waver over time. Engagement is not a well-defined term but can be interpreted as both the experience of completing a task and the participant’s behaviour when interacting with a task or intervention, such as how many sessions an individual completes or the timepoint at which they leave the study [15,18]. Gamification—the process of adding game elements to tasks and systems without actually creating a game [19]—presents a potential avenue to increase engagement with cognitive tasks and training. The rise in studies of gamified and game-like programs and tasks has resulted in several reviews to determine whether gamification can enhance intervention effectiveness, though the findings are mixed. Lumsden *et al*. [15] and Vermeir *et al*. [20] found tentative evidence that gamification can increase motivation and engagement with cognitive tasks, however, whether this translates to increased adherence to an intervention schedule is unclear [15]. Najberg *et al*. [21] achieved protocol adherence rates of 95% with their food-based go/no-go and cued approach training, though there was no non-gamified control group, and the incentive for taking part may have been valuable enough to motivate participants to adhere to the protocol, with incentives known to improve recruitment and adherence rates [22]. Aulbach *et al*. [16] found a sharp decline in the number of participants using a gamified F-RIT app (FoodT) over the first five days of use in an opportunistic study of real-world usage with no incentive on offer. Furthermore, some studies have found that adding gamification elements may actually weaken training effects, which may be explained by game elements creating a distraction from the core stimuli [23]. Careful consideration of how and when to add game-like elements is therefore important, but these elements are rarely examined in isolation [20,24], rendering their effects unclear. Game elements should be chosen from a theoretical perspective [25,26], with self-determination theory [27] commonly used to understand the potential role gamification elements play in increasing motivation and engagement in tasks.

Self-determination theory proposes that three psychological needs drive intrinsic motivation; that is, motivation without a need for external reward. The need for competence refers to the desire to feel success when interacting with an environment; relatedness refers to a desire to belong to a group and feel coherent within a social structure and autonomy refers to the desire to both be free to choose to perform an action and feel performing that action is consistent with one’s personal values [25]. Ryan *et al.* [28] found these constructs predicted future video game-playing behaviour, though whether gamifying otherwise serious tasks affects these motivational constructs is an area needing more research [24]. A survey of gamified work-related apps, such as those used for productivity and task management, found no effect of gamification on measures of autonomy, relatedness or competence [29], though the apps were varied in purpose and nature of the gamified elements. In a review of the computerized cognitive training literature, Vermeir *et al*. [20] found the most common game elements to be those related to achievement and progression, such as point-based systems and feedback loops, and immersion elements such as a story or theme. These elements can be mapped to fulfilling psychological needs as set out by self-determination theory, with Sailer *et al*. [25] finding that specific elements (e.g. points and leader boards) were rated higher on corresponding psychological needs (e.g. competence). However, there remains a paucity of research investigating the effectiveness of single gamified elements [30]. Though recent research has investigated the effects of elements in isolation, as well as when different elements are combined in simple and single session tasks, further work is needed to determine the effects of isolated game elements and whether they lead to changes in intrinsic motivation across more complex and longer interventions [31].

The current study, therefore, aims to examine the effects of isolated gamification elements on F-RIT engagement, adherence and effectiveness in comparison to a well-established non-gamified intervention control. Our first gamified group adds social elements, by allowing participants to pick and join a team to contribute to, which is thought to increase motivation by fulfilling a need for social relatedness and providing a sense of relevance to their completion of the gamified task [25]. Our second gamified group adds feedback elements, thought to increase motivation by addressing a psychological need for competence according to self-determination theory [25]. There are four specific research questions each with associated hypotheses:

— RQ1. Does gamification lead to improved training adherence and training motivation compared to the standard, non-gamified version of F-RIT?

- H1a. The gamified training groups will have a significantly greater number of completed sessions compared to the non-gamified training control group.
- H1b. The gamified training groups will report higher levels of training motivation compared to the non-gamified training control group.

— RQ2. Does gamification improve training effects on food evaluations and snacking?

- H2a. There will be a larger decrease in the liking ratings for unhealthy items in gamified groups compared to the non-gamified training control group.
- H2b. There will be a larger increase in the liking ratings for healthy foods in gamified groups compared to the non-gamified training control group.
- H2c. The gamified training groups will display a greater reduction in unhealthy food item snacking in the week following completion of the training compared to the control group.

— RQ3. Does training motivation and adherence mediate training response?

- H3a. Pre- to post-intervention differences in both unhealthy and healthy food item evaluations will be mediated by training adherence.
- H3b. Pre- to post-intervention differences in both unhealthy and healthy food item evaluations will be mediated by training motivation.
- H3c. Pre- to post-intervention differences in snacking frequency will be mediated by training adherence.
- H3d. Pre- to post-intervention differences in snacking frequency will be mediated by training motivation.

— RQ4. Is there equivalence between the gamification types for training adherence and motivation?

- H4a. Training adherence rates will be equivalent between feedback and social gamified training groups.
- H4b. Training motivation will be equivalent between feedback and social gamified training groups.

Given the lack of previous work on the effect of gamification on specific components of motivation and the potential equivalence of training effectiveness between single task gamification groups, we do not propose to test any hypotheses, however, we do state our intention to explore the effects of gamification here to inform future research.

This project also included measures of stress, personality, dietary behaviours, video game experience and inhibitory control for the purpose of student dissertation projects, but these do not form part of any hypothesis tests or exploratory analysis herein.

## 2. Methods

### 2.1. Transparency and openness statement

All data are publicly available online via the University of Bath data repository archive: https://researchdata.bath.ac.uk/id/eprint/1415, and materials and code are available on the OSF project page for this study: https://osf.io/jdk5f/. This study was given in principle acceptance on 17 November 2023 and the preregistered stage 1 protocol is available at: https://osf.io/jspf3. In the sections below, we report all manipulations, measures and exclusions. This study meets level 6 of the PCI RR bias control (https://rr.peercommunityin.org/help/guide_for_authors). The stage 2 recommendation can be found at: https://rr.peercommunityin.org/articles/rec?id=874.

### 2.2. Ethical statement

This study was given a favourable opinion by the Biomedical Sciences Research Ethics Committee at the University of Bath, with approval number 0260-2006. All participants provided informed consent.

### 2.3. Design

The study utilized a three-arm randomized controlled design, with intervention type as the three-level grouping variable (non-gamified food inhibition training (control), achievement-related gamified inhibition training and social-oriented gamified inhibition training) and pre- and post-intervention as our repeated measures variable. Participants were randomly allocated to groups using block randomization [32], with a block size of 3, and blinded to the other training conditions in the study. Participants were recruited via research participation schemes (e.g. SONA Systems Ltd), Prolific Academic (https://prolific.co/) and social media platforms (e.g. Twitter, Instagram).

### 2.4. Participants

The inclusion criteria for this study were as follows:

— Aged 18–65 with a body mass index (BMI) of 18.5 or above (suggesting a ‘healthy’ weight or above), consistent with previous research investigating F-RIT training (e.g. [7]).
— Participants reported snacking on either crisps, chocolate, biscuits and/or cake (foods high in sugar, salt and fat) at least three times per week, as measured on an unhealthy snacking food frequency questionnaire, consistent with previous work investigating the effect of computerized response inhibition training (e.g. [7]).
— Had access to a stable internet connection and a personal computer or laptop.

The exclusion criteria for this study were:

— A current or previous clinical diagnosis of an eating disorder or diabetes, or self-identifying as having either an eating disorder or diabetes.
— Currently attending a formal weight loss programme or using weight loss medication.
— Currently attempting to quit smoking, due to the changes in appetite and food cravings during nicotine withdrawal [33].

### 2.5. Sample size estimation

Based on our resources, we estimated that it was possible to recruit 80 participants per group, for a sample size of 240 in total. This allowed us to detect an effect size of *f* = 0.23 with 90% power. Given previous literature finding a large effect of gamification on task engagement, *g* = 0.72 (which we approximate to a Cohen’s *f* value of 0.36), with no evidence of publication bias [20], we believe this to be an appropriate target sample size that yields informative results.

To measure our secondary hypotheses, an effect size of *f* = 0.24 was estimated for devaluation scores based on the previous work of the authors (*d* = 0.48; [7]). An *a priori* power analysis (G* power; [34]) indicated that to detect an interaction effect between three groups with two measurements, a total sample size of 60 is required to achieve 90% statistical power.

Finally, from a power analysis using the TOSTER R package [35], we would be able to detect equivalence within the parameters *d* = −0.46 and *d* = 0.46 at 80% power with a sample size of 80 per group. We have been more lenient with our target power in this analysis to target relevant effect sizes that correspond to our previously stated effect size of interest (converting from *f* values of 0.23). Our total target sample size was, therefore, set at 240 participants.

We achieved this sample size with the final sample comprising 252 participants (*M*_AGE_ = 34.94, s.d. = 14.10, 67% female), which had 90% power to detect effects of *f* = 0.22.

### 2.6. Materials and measures

#### 2.6.1. Training conditions

**Figure 1.**
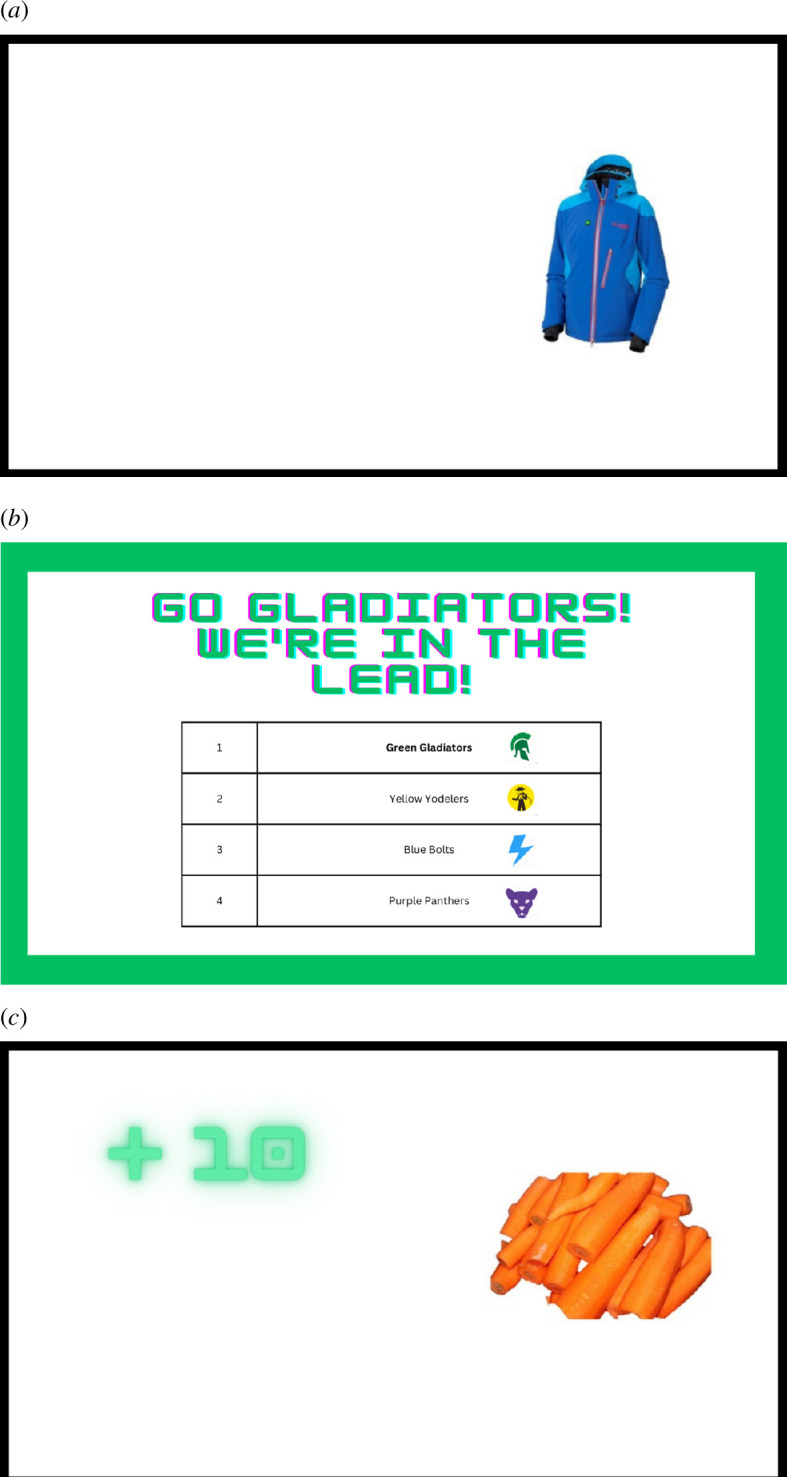
(a) An example of standard non-gamified food RIT control ‘go’ stimulus. (*b*). An example of socially gamified food RIT league table screen. (*c*). An example of feedback gamified food RIT ‘no-go’ correct response points feedback.

A *non-gamified* standard F-RIT was used as our active control group and was taken from Lawrence *et al*. [7]. Pictures of 18 food items, 9 being healthy (e.g. fruit or vegetables) and 9 being high-energy density foods (defined as being greater than 4 kcal g^−1^ such as cakes and chocolate), and 18 non-food items (clothes) were presented on either the left or right of a screen. Stimuli were presented for 1250 ms, followed by a 1250 ms inter-stimulus interval. Participants pressed a key corresponding to the position of the stimuli on the screen (‘c’ for left and ‘m’ for right). Stimuli were presented on a white screen with a frame at the border, which turned bold on trials where the participant was instructed to inhibit their response (‘no-go’ trials, see figure 1*a*). Healthy food items were always paired with the ‘go’ instruction and unhealthy items were always paired with the ‘no-go’ instruction, while non-food items were associated with ‘no-go’ instructions in 50% of trials. Each of the 36 images was presented once per block, with 6 blocks per training session.

The *feedback gamified* F-RIT was identical in nature to the training protocol described previously, with the addition of points awarded or deducted based on the participant’s performance in each block. Ten points were awarded for correct ‘go’ responses and commission errors resulted in a five-point deduction, with visual confirmation of each provided after each trial (see figure 1*c*). At the end of each training session, participants were awarded a badge, decorated with a number of stars corresponding to the number of training sessions in a row they had completed. This manipulation was chosen as it is a frequently included gamification element (e.g. [20,26]).

The *pseudo-socially (social) gamified* F-RIT was again identical in nature to the standard response inhibition training, with the addition of a social element at the beginning and end of each training session. Participants selected a team to join upon enrolment in the group: ‘green’, ‘purple’, ‘yellow’ or ‘blue’. This then became their team for the duration of the training period, the border around the screen signalling a ‘go’ or ‘no-go’ trial changed to their selected team colour around the screen. At the beginning of each training session, participants were informed of their team’s position in a league table (see figure 1*b*). This position increased by one, remained constant or decreased by one at the end of each session. Socially oriented gamified elements are among the least researched elements [20].

#### 2.6.2. Confirmatory outcome variables

*Food liking and value judgement confidence* were measured with a food evaluation task, in accordance with previous research [7]. Twenty-seven pictures of food were presented—the same nine healthy food and nine high-energy density food images that were included in the inhibition training, with the other nine being untrained food images. Participants were asked to rate how much they liked each food image on a 100 mm visual analogue scale, anchored at the extremes of ‘not at all’ (0) and ‘very much’ (100), and then asked how confident they were in the evaluation they just gave. Participants selected their rating on the scale with their mouse cursor, which was reset to the midpoint (a rating of 50) at the start of each trial. Both liking and confidence ratings were collected pre- and post-intervention.

*Snacking frequency* was measured with a snacking food frequency questionnaire (FFQ; 36). This was presented as an 8-item, 7-point Likert-type scale, asking about how frequently different foods (i.e. crisps, cakes, chocolate and biscuits) were consumed over the previous two weeks. Answers ranged from 1 (‘not at all’) to 7 (‘more than four times per week’). Responses were summed to form a total score, with higher scores indicating greater snacking.

*Adherence* was measured as the number of training sessions out of 10 during the 14-day period completed by a participant with an accuracy of >80% on both go and no-go trials.

*Training motivation and enjoyment* were measured by a questionnaire given at the end of each training session. Scores for each questionnaire were summed to give a daily score, with overall training motivation approximated as the mean of all daily scores. The questionnaire was adapted from Lumsden *et al*. [15] and included the following items: ‘1. How enjoyable did you find that?; 2. How frustrating did you find that?; 3. How mentally stimulating did you find that?; 4. How repetitive did you find that?; and 5. How willing would you be to do that again tomorrow?’ Participants recorded their answer to each item on a 100 mm visual analogue scale with ‘not at all’ at one end, to ‘very’ at the other with no subdivisions. After reverse scoring questions 2 and 4, mean item scores were calculated for each participant, with higher values indicating greater subjective enjoyment.

#### 2.6.3. Exploratory outcome variables

*Intrinsic motivation* was assessed by the intrinsic motivation inventory [37] to refer specifically to the training task (see appendix A). This is a 19-item questionnaire with answers ranging from 1 (‘not all’) to 7 (‘very true’) with items measuring relatedness (e.g. ‘I felt connected with the others taking part in this study’), autonomy (e.g. ‘I believe I had some choice about completing each training session’) and competence (‘I think I did pretty well at this training, compared to other participants’). After reverse scoring relevant items, scores were summed to create total scores for each subscale, with higher scores indicating a greater sense of relatedness, autonomy and competence.

*Lifestyle factors* were assessed with a 100 mm visual analogue scale as before, set at the midpoint (a value of 50), anchored at either end with ‘no’ (0) and ‘yes’ (100). Questions asked about a participant’s sleep quality from the previous night (‘Did you get enough sleep last night?”), whether they were currently hungry (‘Are you currently hungry?”) and whether they were hungover (‘Are you currently hungover’) were shown after each training session.

### 2.7. Attention checks

To protect against careless responding [38] participants were asked the multiple-choice question, ‘What planet do you live on?’ with the available response options of: ‘Earth, Mars, Mercury, Saturn’ after the food evaluation task (taken from [39]). Analyses were conducted with and without participants who failed this attention check.

### 2.8. Procedure

We used the Gorilla Experiment Builder (**www.gorilla.sc**) to create and host our experiment [40].

Potential participants first completed a screening stage consisting of completing the FFQ [36]. They were also asked their height and weight, and this was then converted to yield their BMI (kg m^−2^). Data collection occurred over two testing sessions: baseline and post-intervention. Heights below 122 cm and above 213 cm (48 and 84 inches), or weights below 34 kg and above 227 kg (75 and 500 pounds) were deemed implausible, and therefore not included in any descriptive statistics [41]. At baseline, we recorded participants’ self-reported weight, food evaluations and snacking frequency, alongside measures of stress, personality, dietary behaviours and inhibitory control for student dissertation projects. Between the two testing sessions, participants were asked to complete 10 sessions of the online training intervention over 14 days. Finally, participants completed all measures again post-intervention. Participants were paid £6 per hour, in line with the guidelines of Prolific Academic and the Lead institution.

### 2.9. Planned statistical analyses

#### 2.9.1. Data exclusion criteria

Outliers on outcome measures were retained for analyses and participants who completed ewer than 10 training sessions were retained in order to achieve an unbiased effect estimate of the intervention [42]. Participants who averaged below 80% accuracy on training sessions or whose accuracy score was greater than 2 standard deviations higher than the group mean were excluded from the analysis. Missing data were assessed for randomness using Little’s test [43], and a missing dummy variable was created and tested for associations with group type.

#### 2.9.2. Planned analyses

All analyses were carried out in R Studio, with details of all packages used included in the electronic supplementary materials. The null hypothesis was rejected when *p *< 0.05, or when *p *> 0.05 for equivalence tests.

##### 2.9.2.1. RQ1: Does gamification lead to improved training adherence and motivation compared to non-gamified training?

All hypotheses relating to this question were tested with a one-way factorial analysis of variance (ANOVA) with training group as the factor, and mean motivation score and the number of training sessions completed as the two dependent variables.

##### 2.9.2.2. RQ2: Does gamification improve training effects for food evaluations and snacking?

All hypotheses addressing this research question were tested with a series of 3 (control, social gamification and reward gamification) × 2 (time, pre–post) mixed design ANOVA tests. Significant main effects were followed up with an analysis of simple main effects to investigate the direction of the effect found.

##### 2.9.2.3. RQ3: Does motivation and adherence mediate training response?

Mediation analyses were conducted using a causal steps approach, as suggested by Hayes & Rockwood [44], for each hypothesis separately. The significance of the mediation model was determined using the bootstrap method, based on 5000 bootstrap samples (consistent with the recommended number from [45]. Mediation coefficients were established with separate regressions: first with the intervention group entered as a predictor (dummy coded with the control set as the reference category) and the change in food item evaluations and in unhealthy food snacking frequency as the two outcome variables. Second, the direct effect of the intervention group on change in training engagement or motivation was established, followed by establishing the indirect effect with both intervention group and change in the mediator as our predictor variables and change in food evaluation score and snacking frequency as our two outcome variables. For all models, baseline scores of our variables were entered as covariates and to establish the significance and confidence intervals, the R package ‘*mediation*’ was used [46].

##### 2.9.2.4. RQ4: Is there equivalence between the gamification types for training adherence and motivation?

All hypotheses for this research question were tested with two one-sided *t*-tests, using the R package *TOSTER* [47]. Equivalence was concluded when the 95% confidence intervals fell between *d =* −0.46 and *d =* 0.46.

### 2.10. Planned exploratory analysis

While not formally testing a specific hypothesis, we also investigated whether gamification resulted in fewer outliers (scores greater than 2 standard deviations from the group mean) during training performance. Second, we explored whether there were differences between the training groups on measures of perceived competence, autonomy and relatedness, in line with Sailer *et al*.’s [25] categorization of gamification elements with self-determination theory. Third, we explored whether gender moderates the relationship between training effects and adherence rates based on previous findings that suggest gender moderates training efficacy in a largely theme-based gamification task [48]. Finally, we explored relationships between the change in food item evaluation confidence, food liking ratings and training adherence.

## 3. Results

### 3.1. Participants

Participant characteristics and descriptive statistics are presented in table 1. One participant failed the attention check and was excluded from all analyses. Pre-registered analyses with this participant included were consistent with the reported analyses below and are included in the electronic supplementary materials. Consistent with our registered plan, for RQs 2 and 3, and all exploratory analyses investigating food item ratings and snacking frequency, we excluded 51 participants who scored less than 80% accuracy average across all training sessions, and 9 participants who did not complete a single training session. Analyses conducted with these participants are included and reported in the electronic supplementary materials. Little’s test suggested data were missing completely at random, χ^2^ = 0, *p* = 1.0.

**Table 1.** Participant characteristics and descriptive statistics (means and s.d.) for all main outcome variables as a function of the training group. BMI = body mass index. FFQ = food frequency questionnaire.

		training group		
variable		control (*n* = 85)	feedback (*n* = 85)	social (*n* = 81)
age		34.57 (13.78)	34.86 (14.18)	35.74 (14.51)
*N* female (%)		56 (66)	59 (69)	54 (67)
baseline BMI^a^		26.50 (5.60)	27.39 (6.11)	26.64 (5.57)
weight (kg)	pre-training	75.47 (16.47)	77.56 (19.19)	76.18 (17.24)
	post-training	73.17 (20.15)	73.34 (22.22)	77.15 (18.01)
FFQ^b^	pre-training	25.27 (8.45)	28.64 (7.93)	25.62 (7.50)
	post-training	23.57 (7.31)	24.52 (8.28)	23.76 (7.37)
healthy food liking	pre-training	49.28 (18.00)	50.87 (17.27)	52.61 (17.33)
	post-training	55.45 (17.45)	56.38 (14.51)	60.12 (15.30)
unhealthy food liking	pre-training	61.61 (20.02)	68.01 (17.53)	58.20 (23.82)
	post-training	65.21(18.93)	69.03 (19.07)	65.19 (20.96)
mean training sessions		3.09 (3.32)	3.40 (3.65)	3.16 (3.76)
*n* > 1 training session (%)		42 (49.4)	45 (52)	30 (37.0)
average daily motivation		49.31 (19.78)	50.01 (19.36)	54.47 (16.94)

### 3.2. RQ1: Does gamification lead to improved training adherence and training motivation compared to the standard, non-gamified version of food response inhibition training?

One-way factorial ANOVAs were conducted in accordance with our registered analysis plan. There were no significant differences between training groups on the number of training sessions completed, *F*_2, 248_ = 0.17, *p* = 0.848, *η_p_^2^* = 0.001, or on mean motivation scores *F*_2, 214_ = 1.40, *p* = 0.250, *η_p_^2^* = 0.012, failing to support either H1a and H1b.

### 3.3. Unplanned exploratory analysis: exploring the effect of the recruitment method

Given that the adherence data are a count variable, and with evidence of overdispersion, a negative binomial regression was also conducted, exploring the effect of including the recruitment method (research participation schemes, Prolific Academic or social media platforms) as a predictor (table 2). There was a significant effect of being recruited through Prolific compared to the social media (exp(*b*) = 0.64, *p* < 0.001, 95% CI [0.47, 0.83]) and university participation panels (exp(*b*) = 0.45, *p* = 0.002, 95% CI [0.28, 0.74]) on adherence rates. This suggests that those recruited through Prolific or university participation schemes were less likely to adhere to the training than those recruited through the social media. There was still no significant effect of the training group and no interactions between the training group and the recruitment method. Given this effect, we determined that the recruitment method should be included as a covariate in our analyses for RQ2 and RQ3, and so report outcomes for our registered hypothesis tests both with and without it.

**Table 2.** Results of negative binomial regression of training group and recruitment method on adherence.

	coefficient	s.e.	*p*	LCI	UCI
control versus feedback group	1.13	0.15	0.413	0.84	1.52
control versus social group	1.06	0.15	0.705	0.76	1.43
**general versus prolific recruitment**	**0.64**	**0.14**	**0.001**	**0.49**	**0.83**
**general versus university RPS recruitment**	**0.45**	**0.25**	**0.002**	**0.28**	**0.74**

In summary, we found no evidence that adding either feedback or social game elements improved adherence or motivation compared to a standard F-RIT training.

### 3.4. RQ2: Does gamification improve training effects on food evaluations and snacking?

**Figure 2.**
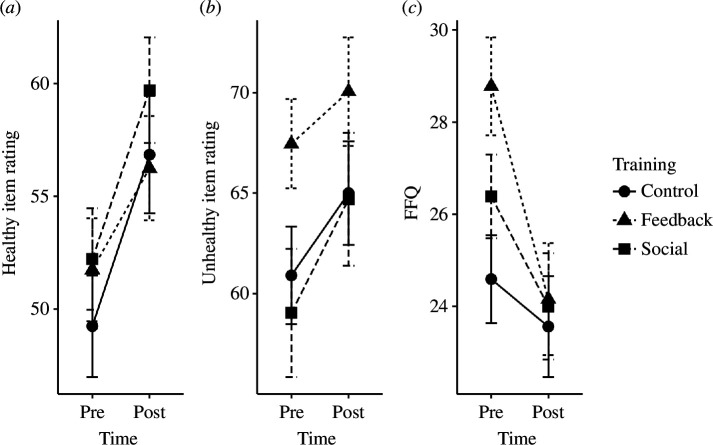
Line graphs of food item ratings and snacking behaviour pre- and post-training.

A series of mixed 3 (group: control, feedback and social) × 2 (time: pre–post training) ANOVAs were conducted for healthy and unhealthy food liking ratings and food item snacking frequency, with results presented in table 3. All distributions met parametric assumptions, as assessed by Shapiro–Wilk tests (all *p* > 0.05). There was a significant main effect of time for each outcome, indicating that participants’ ratings for both healthy, *F*_1, 125_ = 41.67, *p* < 0.001, *η_p_^2^* = 0.260 and unhealthy food items, *F*_1, 125_ = 4.76, *p* = 0.03, *η_p_^2^* = 0.039 increased from pre- to post-training, as well as a reduction in snacking frequency, *F*_2, 125_ = 20.52, *p* < 0.001, *η_p_^2^* = 0.141. There was no significant group × time interaction effect on either healthy or unhealthy food item liking ratings, and therefore we found no support for either H2a or H2b (see figure 2). Healthy and unhealthy food item confidence ratings did not change significantly over time, nor was an interaction effect found, with these results presented in the electronic supplementary materials.

**Table 3.** Results of mixed 3 (group) × 2 (time) ANOVAs for food item ratings and snacking behaviour.

	*F*	*p*	*η_p_^2^*
healthy food item ratings			
group	0.35	0.707	0.006
**time**	**41.67**	**<0**.**001**	**0**.**260**
group × time	0.46	0.632	0.009
unhealthy food item ratings			
group	1.11	0.334	0.017
**time**	**4.76**	**0**.**031**	**0**.**039**
group × time	0.40	0.987	<0.001
snacking behaviour			
group	2.14	0.122	0.024
**time**	**20.52**	**<0**.**001**	**0**.**041**
**group × time**	**3.84**	**0**.**024**	**0**.**016**
healthy rating confidence			
group	0.26	0.770	0.004
time	0.13	0.711	0.001
group × time	0.59	0.555	0.009
unhealthy rating confidence			
group	0.28	0.756	0.004
time	1.86	0.176	0.017
group × time	0.75	0.471	0.015

Bold denotes *p* < 0.05.

There was a significant two-way interaction between group and time for snacking frequency *F*_2, 125_ = 3.84, *p* = 0.024, *η_p_^2^* = 0.054. Follow-up simple main effects indicated a significant effect of group at baseline after correcting for multiple comparisons, *F*_2, 188_ = 4.64, *p* = 0.022, *η_p_^2^* = 0.047, but no significant effect of group at the post-intervention testing session, *F*_2, 125_ = 0.07, *p* > 0.999, *η_p_^2^* = 0.001. Pairwise comparisons indicated that pre-training FFQ scores for the feedback group were significantly higher than the control group (*p* = 0.008), but no other comparisons were significant, suggesting baseline differences drove this interaction effect, thus failing to support H2c.

### 3.5. Unplanned exploratory analysis: including recruitment method as a covariate

Including the recruitment method as a covariate changed several results. There was no longer a significant main effect of time for healthy food item ratings, *F*_1,123_ = 2.10, *p* = 0.149, *η_p_^2^* = 0.002; unhealthy food item ratings, *F*_1,123_ = 0.02, *p* = 0.883, *η_p_^2^* = 0.001; or snacking frequency, *F*_1,123_ = 0.43, *p* = 0.513, *η_p_^2^* = 0.001. As before, there was no significant main effect of group, and the group × time interaction remained significant for snacking frequency, *F*_2, 123_ = 4.07, *p* = 0.020, *η_p_^2^* = 0.02, again suggesting baseline group differences drove this interaction. No other group × time interactions were significant. There was also a significant main effect of the recruitment method for healthy food item ratings, *F*_2, 123_ = 3.97, *p* = 0.021, *η_p_^2^* = 0.05; and snacking frequency, *F*_2, 123_ = 13.60, *p* < 0.001, *η_p_^2^* = 0.14. Finally, there were no significant interactions between time and recruitment method for unhealthy food item ratings, *F*_2, 123_ = 1.45, *p* = 0.237, *η_p_^2^* = 0.002 and snacking frequency, *F*_2, 123_ = 2.27, *p* = 0.108, *η_p_^2^* = 0.01, but there was a significant interaction for healthy food item ratings, *F*_2, 123_ = 5.19, *p* = 0.007, *η_p_^2^* = 0.01. Paired *t*-tests for each recruitment method found that participants recruited through Prolific Academic showed a significant increase in healthy item food ratings from pre- (*M* = 47.15, s.d. = 18.16) to post-training (*M* = 56.18, s.d. = 16.61), *t*(84) = −6.93, *p* < 0.001, *d =* −0.75 but there were no significant differences for the other two recruitment methods.

These results suggest the group × time interaction for snacking frequency is robust, and healthy item ratings and snacking frequency differed between the recruitment streams.

### 3.6. Unplanned exploratory analysis: training manipulation check and the effect on weight

Consistent with previous research on F-RIT (e.g. [7]), we looked for evidence of a stimulus–response relationship during the training sessions. Reaction times for ‘go’ trials were significantly quicker for healthy food (*M* = 858.43 ms, s.d. = 69.89 ms) than filler images (*M* = 872.94 ms, s.d. = 77.97 ms), *t*(184) = 5.46, *p* < 0.001, *d* = 0.40, which is consistent with participants learning to respond (‘go’) to healthy foods. There were no significant differences in commission errors between filler (*M* = 0.97, s.d. = 0.04) and unhealthy (*M* = 0.97, s.d. = 0.03) food no-go trials, *t*(184) = −1.75, *p* = 0.082, *d =* −0.13 which may suggest weak learning effects to inhibit responses to unhealthy foods.

A linear regression investigating the effect of both the training group and the recruitment method on the overall training accuracy found no significant effects (see electronic supplementary materials for model parameters), suggesting there were no performance differences between the groups, and participants from all recruitment methods performed similarly during the training task.

Finally, we conducted a mixed 3 (group) × 2 (time) ANOVA to investigate the effect on participants’ self-reported weight. There was a significant main effect of time, *F*_2, 121_ = 4.03, *p* = 0.046, *η_p_^2^* = 0.03, indicating that participants’ weight decreased across the training period, but there was no significant main effect of group or two-way interaction suggesting that gamification did not influence weight change.

In summary, we found that adding feedback or social game elements did not accentuate changes in healthy or unhealthy food item evaluations, but those assigned to the feedback group experienced a greater decrease in snacking behaviour.

### 3.7. RQ3: Does training motivation and adherence mediate training response?

Coefficients for each step in the mediation models are presented in table 4. Bootstrapped confidence intervals found no significant mediating effect of training adherence or motivation scores on the baseline to post-intervention differences for healthy or unhealthy food liking judgements or change in snacking frequency, thus contrary to H3a, H3b, H3c and H3d. However, two direct effects in the regression models were of note: first, assignment to the feedback group, as compared to the control, predicted a greater reduction in snacking frequency, *b* = −4.54, *p* = 0.007, 95% CI [−7.71,−1.26]. Second, adherence to training predicted a decrease in unhealthy food liking judgements, *b* = −0.70, *p* = 0.015, 95% CI [−1.26,−0.14].

**Table 4.** Parameter estimates for mediation models.

	model	estimate (s.e.)	LCI	UCI	*p*
feedback versus control	adherence	0.15 (0.87)	−1.58	1.88	0.866
social versus control	adherence	0.46 (0.88)	−1.28	2.19	0.603
feedback versus control	motivation	3.51 (4.03)	−4.47	11.49	0.386
social versus control	motivation	7.67 (4.10)	−0.45	15.80	0.064
adherence	Δ healthy ratings	0.04 (0.28)	−0.51	0.59	0.898
feedback versus control	Δ healthy ratings	−2.00 (2.71)	−7.37	3.36	0.462
social versus control	Δ healthy ratings	0.37 (2.73)	−5.03	5.78	0.892
indirect effect		0.01	−0.51	0.97	.97
**motivation**	**Δ healthy ratings**	**0.17 (0.06)**	**0.05**	**0.29**	**0.006**
feedback versus control	Δ healthy ratings	−2.51 (2.69)	−7.83	2.82	0.353
social versus control	Δ healthy ratings	−1.23 (2.77)	−6.76	4.20	0.645
indirect effect		0.60	−0.86	2.48	0.39
**adherence**	**Δ unhealthy ratings**	**−0.70 (0.28)**	**−1.26**	**−0.14**	**0.015**
feedback versus control	Δ unhealthy ratings	0.53 (2.76)	−4.94	6.00	0.848
social versus control	Δ unhealthy ratings	0.67 (2.78)	−4.84	6.17	0.810
indirect effect		−0.10	−1.45	1.14	0.85
motivation	Δ unhealthy ratings	−0.04 (0.06)	−0.17	0.09	0.506
feedback versus control	Δ unhealthy ratings	0.25 (2.87)	−5.44	5.94	0.890
social versus control	Δ unhealthy ratings	−0.41 (2.96)	−6.27	5.45	0.890
indirect effect		−0.11	−0.87	0.99	0.88
adherence	Δ snacking	−0.08 (0.17)	−0.42	0.25	0.618
**feedback versus control**	**Δ snacking**	**−4.54 (1.66)**	**−7.81**	**−1.26**	**0.007**
social versus control	Δ snacking	−2.72 (1.67)	−6.02	0.58	0.105
indirect effect		−0.01	−0.35	0.36	0.92
motivation	Δ snacking	−0.03 (0.04)	−0.11	0.04	0.419
**feedback versus control**	**Δ snacking**	**−4.69 (1.70)**	**−8.05**	**−1.33**	**0.007**
social versus control	Δ snacking	−2.81 (1.66)	−6.04	0.87	0.141
indirect effect		−0.11	−0.65	0.36	0.71

### 3.8. Unplanned exploratory analysis: including recruitment method as a covariate

When including the recruitment method in the models, outcomes for the tests of mediation effects were unchanged, and support for the effect of motivation on the change in healthy item ratings (see table 4) remained, as did the effect of assignment to the feedback group on change in snacking frequency, *b* = −4.51, *p* = 0.005, 95% CI [−7.64,−39]. There were some differences in the models, namely the effect of assignment to the social gamification group on mean daily motivation scores met the significance threshold, *b* = 9.55, *p* = 0.016, 95% CI [1.78, 17.32], while the effect of training adherence on change in unhealthy item ratings instead suggested only weak evidence for this effect, *b* = −0.53, *p* = 0.063, 95% CI [−1.09, 0.02].

In summary, we found no evidence that adherence or motivation mediated the relationship between training group and change in healthy ‘liking’ ratings, unhealthy ‘liking’ ratings or snacking behaviour. We again found assignment to the feedback group predicted a greater change in snacking behaviour, and additionally found that greater adherence to the training predicted a decrease in unhealthy food item ratings.

### 3.9. RQ4: Is there equivalence between the gamification types for training adherence and motivation?

Two one-sided *t*-tests found that training adherence rates, *t*(164) = −.40, *p* = 0.345, *g =* 0.06 and mean daily motivation scores, *t*(164) = 1.41, *p* = 0.920, *g =* 0.24, were statistically equivalent between the two groups, suggesting there were no differences equivalent to or larger than *d =* 0.46 in these two outcomes between the gamification types.

### 3.10. Pre-registered exploratory analyses

Full results of our pre-registered exploratory analyses are presented in the electronic supplementary materials, though we present the main findings here.

A binomial logistic regression investigating the effect of the training group on whether a participant fell 2 standard deviations away from their group training accuracy mean was not significant χ^2^ (2) = 1.59, *p* = 0.451. This suggested that gamification did not change training performance.

Linear regression models were conducted to explore the effect of gamification on intrinsic motivation, as defined by Sailer *et al*.’s [25] categorization of gamification elements according to self-determination theory. There was evidence of an effect of adding feedback elements on a measure of perceived autonomy at completing the training tasks, *b* = 1.90, *p* = 0.047, 95% CI [0.03, 3.78], though there was no effect of gamification on measures of perceived competence or relatedness, *p* > 0.05.

Given there was only an effect of adherence on change in unhealthy food item liking ratings, as determined in our analyses for RQ3, a moderated regression investigating the interaction between gender and adherence was only conducted with change in unhealthy food item liking ratings as an outcome. As in Forman *et al*. [48], we restricted our gender variable to men and women for this analysis. Adherence data were centred, and there was no significant evidence of an interaction between gender and adherence to the training, *b* = 0.20, *p* = 0.737, 95% CI [−0.99, 1.40].

We explored the relationship between changes in food item liking ratings, rating confidence and training adherence with Spearman’s rank correlations given the non-normal distribution of our adherence data (see table 5). Changes in unhealthy food confidence ratings were positively associated with a change in the liking rating of that item (*rho* = 0.21, *p* = 0.015), suggesting as individuals were less confident in their ratings, their liking of those items reduced. The change in unhealthy item ratings was also positively associated with the change in healthy item ratings (*rho* = 0.21, *p* = 0.010), suggesting that changes in endorsement of these items occurred in tandem. There were no other significant relationships between these variables, all *p > *0.05.

**Table 5.** Correlation analyses between food liking ratings, rating confidence and training adherence.

	coefficient	*p*	LCI	UCI
Δ healthy ratings − Δ healthy ratings confidence	0.05	0.561	−0.09	0.19
Δ healthy ratings − adherence	0.01	0.873	−0.13	0.16
**Δ unhealthy ratings − Δ unhealthy ratings confidence**	**0.21**	**0.015**	**0.08**	**0.35**
Δ unhealthy ratings − adherence	−0.15	0.090	−0.29	0.01
**Δ healthy ratings − Δ unhealthy ratings**	**0.21**	**0.010**	**0.07**	**0.33**
Δ snacking − adherence	0.07	0.413	−0.07	0.21
Δ snacking − Δ healthy ratings	−0.07	0.436	−0.21	0.07
Δ snacking − Δ unhealthy ratings	−0.03	0.754	−0.17	0.11

### 3.11. Unplanned exploratory analysis: the influence of lifestyle factors on training performance and engagement

Finally, we explored the relationship between lifestyle factors, daily training accuracy and daily motivation with Pearsons’s correlations (see table 6). Daily motivation was positively associated with training accuracy (*r* = 0.19, *p *< 0.001), but no other significant relationships were present, all *p *> 0.05.

**Table 6.** Results of correlation analyses between lifestyle factors, daily training accuracy and daily motivation.

	coefficient	*p*	LCI	UCI
hunger − accuracy	−0.05	0.209	−0.12	0.03
tiredness − accuracy	0.07	0.068	−0.00	0.14
hungover − accuracy	0.01	0.683	−0.06	0.09
**daily motivation − accuracy**	**0.19**	**<0.001**	**0.13**	**0.26**
hunger – daily motivation	0.02	0.659	−0.06	0.09
tiredness – daily motivation	0.05	0.262	−0.03	0.11
hungover – daily motivation	0.03	0.430	−0.04	0.10

## 4. Discussion

With little research investigating the effect of single gamified elements on engagement motivation for computerized cognitive interventions [31], and given the calls for freely available software to gamify experiments [49], we used Gorilla, a widely used experiment builder with an integrated game builder, to investigate the effect of isolated game elements (feedback or social) on adherence, motivation and the effectiveness of computerized F-RIT. Contrary to our hypotheses, we found no statistically significant evidence suggesting that the addition of isolated game elements (feedback or social) influenced either training adherence or daily motivation compared to a non-gamified control intervention. We found a reduction in snacking frequency across the training period for all groups. We also found liking ratings increased for both healthy and unhealthy food items from pre- to post-training, the latter of which was against our predictions.

Against our main hypothesis, adding isolated game elements did not significantly improve training adherence and motivation. While the two gamified groups exhibited slightly higher adherence and motivation scores on average, this was not significantly different from the control group. There was weak evidence for an effect of assignment to the social gamification group on daily training motivation, though this met significance criteria only after adjusting for the recruitment method. These results are somewhat consistent with previous reviews suggesting that gamified tasks may be more engaging or motivating than non-gamified tasks [15,20], but this does not appear to translate into improved adherence rates (e.g. [15]). Given the evidence for equivalence between the two gamification groups on measures of adherence and motivation and lack of effect in our registered analyses, it is probable that isolated game elements may produce effects too small (*f*^2^ = 0.04 for adherence and *f*^2^ = 0.11 for motivation) to be detected with our sample size. Our sample size estimation was informed by previous work finding large effects of gamification [20], with previous research mainly implementing multiple gamified elements. Single element gamification may, therefore, produce effects smaller than we could detect, however, as even small effect sizes in computerized interventions may be meaningful [50], future research investigating what constitutes a meaningful effect size for adherence and motivation would be a valuable addition to the literature.

We selected feedback and social elements based on Sailer *et al*.’s [25] classification of gamified elements into measures of intrinsic motivation: competence, relatedness and autonomy. In exploratory analyses, we found that participants assigned to the feedback group displayed significantly higher levels of perceived autonomy; that is, they felt it was their choice to complete training sessions rather than something they felt obliged to do as part of the study. However, there were no significant differences between the groups on the expected concepts of competence or relatedness. The social gamified elements aimed to recreate social elements described by Sailer and colleagues to foster a sense of relatedness and obligation to others through picking a team, the colour scheme of the programme adapting to that theme and a competitive element through a leaderboard. However, given that there was no real social interaction with other members of the team, it may be that these social elements were not effective. As social elements are among those rarely implemented and measured [20], further research is required on how to best implement them.

There was also a lack of evidence that adding feedback elements increased feelings of competence at completing the training, against expectations. Unlike adding social elements, there is precedence for each of the feedback elements in isolation, with Lumsden *et al*. [15] adding a points variation to a similar task relying on motor inhibition. They found participants in the points variant group had the highest average enjoyment rating of all conditions; however, we found no such effects, either in daily motivation or in the intrinsic motivation measure completed in the post-training testing session. Our findings are, however, consistent with that of Mekler *et al*. [51] who found no effect of gamification on measures of intrinsic motivation. Nevertheless, while self-report measures of motivation and enjoyment showed no differences, those assigned to the feedback group had a higher average accuracy on the training task in our exploratory analysis, suggesting improved performance while completing the task.

Recreational computer games are likely intrinsically motivating as players find themselves in a cycle of being presented with a challenge and expending effort to surmount that challenge [52]. The F-RIT task in this study may not have been challenging enough to induce a feeling of competence or achievement in having completed the task with a high accuracy rate, given the consistent ratio of ‘go’ to ‘no-go’ stimuli. Previous research suggests that the addition of gamified elements is unlikely to produce any additional benefit if the task is not sufficiently challenging [51].

Taken together, our findings may suggest an improved engagement with the training task while it was being performed, though this does not relate to motivation to complete the task. This has implications for gamification of non-adaptive cognitive tasks, such as F-RIT, which often requires a consistent pairing of healthy images with ‘go’ responses and unhealthy images with ‘no-go’ responses. Such training may benefit more from other gamified elements that either improve the visual appeal of the training or increase task complexity.

Our hypotheses that gamification would accentuate changes in healthy (H2a) and unhealthy (H2b) food liking ratings were also not supported. Instead, ratings for healthy and unhealthy items increased across the training period for all three groups. We had expected ratings for unhealthy foods to decrease over time, consistent with many studies demonstrating devaluation effects for trained no-go foods [4,12]. Indeed, we observed an association between greater training adherence and the expected devaluation of unhealthy foods here, aligning with previous reports that more training is associated with greater reductions in liking and intake of unhealthy no-go foods [16], including a study very similar to this one [53]. It may be that the lack of overall unhealthy food devaluation resulted from a lack of robust learning of unhealthy food-no-go associations during training (our manipulation check failed to demonstrate such learning effects) because previous research suggests that attention to and memory (awareness) of food-no-go contingencies is an important determinant of reductions in liking and preference [54, 55].

Rather, our findings suggest that participants showed stronger learning of the healthy food-go association, which can increase food liking [55], and this may have generalized to all foods. Another potential reason for the lack of devaluation effects was the long time lag between the final session they completed (e.g. if they stopped training after the second day) and the next evaluation of food items (e.g. day 15). Evidence suggests that training-related increases in preference for ‘go’ foods are more robust and last longer than decreased preference for ‘no-go’ foods, although the latter has been shown to last for up to one week [55]. Nevertheless, caution is required in interpreting the present increased ratings of foods over time, given this disappeared after including the recruitment method as a covariate. Our exploratory analysis found that participants recruited via Prolific Academic increased their liking ratings for all food items, even untrained foods, with no differences found in those recruited by other methods. It may be that Prolific participants are more experienced in completing cognitive tasks and adopted a different strategy during the training, e.g. only attending to the ‘go’ signals or trials. We did not use any implicit measures of food evaluations in this study (as these are less sensitive to no-go training effects than explicit evaluations [12]), though it may be interesting to include such measures in future research to help interpret any unexpected changes in explicit evaluations, such as the generalized increase in food liking seen in Prolific Academic participants here.

Consistent with previous research, snacking frequency decreased for all groups. This may suggest that the training worked as expected in reducing consumption of no-go foods (albeit without their devaluation) or it may reflect general effects of participation, such as expectancy effects. For example, participant expectations about the intervention may affect their behaviour or responses, or self-monitoring may increase as a result of answering questions about their diet [4]. This may also explain the reduction in weight across all groups. Compared to the control group, participants assigned to the feedback group showed significantly lower snacking frequency (consistent with H2c), but we are cautious about over-interpreting this result given it was not mediated by adherence or motivation (H3c) and may be due to baseline differences.

In summary, we did not find support for our hypotheses that gamification would influence training effects, though we cannot make further inferences as to whether gamification may improve or hinder training effects as in [23] given the generally poor adherence to the intervention.

### 4.1. Strengths, limitations and future directions

One strength is that our study did not incentivize the completion of the training sessions. Incentives have been shown to improve adherence rates [22], potentially conflating the effect of training with the monetary reward. Our findings, therefore, offer a more accurate estimate of the effect of implementing single gamified elements in F-RIT on training adherence and motivation. Another strength was the inclusion of a non-gamified control group, which allowed us to isolate any additive effect of gaming elements as well as address the dearth of non-gamified control groups in previous gamification research [20,56]. Finally, our study addresses previous issues identified in this field, such as low statistical power in response inhibition training studies [57] and low study quality in the gamification literature [20], by conducting this study through the Registered Report publication model. Such a model has been found to reduce publication bias (58) and improve research quality compared to non-registered reports [59]. As such, this study may provide more reliable effect size estimates for this research field and contribute to meta-analytic tests of the utility of gamification in response inhibition training.

There are, however, several limitations which can inform future research in this area. First, we recruited participants from a variety of sources, namely Prolific Academic, university research participation panels and generally through researcher adverts, contact lists and social media. These groups differed in their demographics (e.g. BMI), and adherence, which required us to conduct exploratory analyses to assess the influence of such recruitment procedures. Future research should either account for this in any pre-specified analysis plans or seek to recruit with only one method; yet with this latter recommendation, researchers should acknowledge that their participant sample is likely to differ based on their chosen recruitment method, which may affect generalizability.

Although we screened our sample for relevant characteristics such as snacking frequency and implemented strict exclusion criteria to ensure those taking part in weight loss interventions did not take part, we did not specify any dieting intentions as part of our inclusion criteria. The majority of previous research in this field has been conducted in a motivated sample (e.g. those wishing to reduce unhealthy eating behaviours or with a desire to lose weight, with some studies not offering any incentives), and it may be that, although our results are appropriate for a casual user, they are not representative of those who are most likely to utilize F-RIT. Capturing participant willingness or desire to either change their diet or lose weight would be a useful addition to future research. App-based F-RIT shows promise as an accessible method of delivery [16] and would represent a logical next step when exploring the effect of gamification in this field. Apps provide other means of increasing adherence, such as reminders and notifications and are instantly accessible through mobile devices, yet research finds relatively low adherence rates (e.g. [16]), and so may benefit from the addition of multiple game elements. Finally, single gamified elements may produce effects too small to be detected in our sample size. However, future research should systematically investigate combinations of game elements to identify the most optimal gamified intervention.

## 5. Conclusions

This study set out to investigate whether single element gamification improved adherence and motivation to F-RIT and whether these may mediate the training’s effectiveness. We found no meaningful evidence for the effect of adding single game elements on food-RIT adherence or motivation. There was also a lack of evidence that gamification alters training effects (e.g. food liking ratings and snacking behaviour), which may be explained by the generally poor adherence rates across the sample. With a view to increasing adherence and motivation for cognitive interventions, such as F-RIT, we recommend that future research increases the challenge or difficulty of the task used, tailor the recruitment method to motivated samples who are likely to benefit from such interventions and consider the impact of different recruitment methods on the measured outcomes.

## Data Availability

All data are publicly available online via the University of Bath data repository archive: https://researchdata.bath.ac.uk/id/eprint/1415, and materials and code are available on the OSF project page for this study: [60]. Supplementary material is available online [61].
